# Eating disorders and physical multimorbidity in the English general population

**DOI:** 10.1007/s40519-023-01600-0

**Published:** 2023-09-07

**Authors:** Lee Smith, Guillermo F. López Sánchez, Emilio Fernandez-Egea, Tamsin Ford, Christopher Parris, Benjamin R. Underwood, Laurie Butler, Yvonne Barnett, Mike Trott, Ai Koyanagi

**Affiliations:** 1 Centre for Health Performance and Wellbeing, Anglia Ruskin University, Cambridge, UK; 2 Division of Preventive Medicine and Public Health, Department of Public Health Sciences, School of Medicine, University of Murcia, Murcia, Spain; 3 Department of Psychiatry, University of Cambridge, Cambridge, UK; 4 Cambridgeshire and Peterborough NHS Foundation Trust, Fulbourn Hospital, Fulbourn, Cambridge, UK; 5 Medical Technology Research Centre, School of Life Sciences, Anglia Ruskin University, Cambridge, UK; 6 Centre for Public Health, Queens University Belfast, Belfast, UK; 7 Research and Development Unit, Parc Sanitari Sant Joan de Déu, Sant Boi de Llobregat, Barcelona Spain

**Keywords:** Eating disorder, Disordered eating, Chronic disease, Multimorbidity, UK, Adults, Epidemiology

## Abstract

**Purpose:**

People with eating disorders may be at increased risk for physical health problems, but there are no data on the relationship between eating disorders and physical multimorbidity (i.e., ≥ 2 physical conditions) and its potential mediators. Thus, we investigated this association in a representative sample of adults from the UK, and quantified the extent to which this can be explained by various psychological and physical conditions, and lifestyle factors.

**Methods:**

Cross-sectional data of the 2007 Adult Psychiatric Morbidity Survey were analyzed. Questions from the five-item SCOFF screening instrument were used to identify possible eating disorder. Respondents were asked about 20 physical health conditions. Multivariable logistic regression and mediation analysis were conducted.

**Results:**

Data on 7403 individuals aged ≥ 16 years were analyzed [mean (SD) age 46.3 (18.6) years; 48.6% males]. After adjustment, possible eating disorder was associated with 2.11 (95%CI = 1.67–2.67) times higher odds for physical multimorbidity. Anxiety disorder explained the largest proportion this association (mediated percentage 26.3%), followed by insomnia (21.8%), perceived stress (13.4%), depression (13.1%), obesity (13.0%), and alcohol dependence (4.3%).

**Conclusion:**

Future longitudinal studies are warranted to understand potential causality and the underlying mechanisms in the association between eating disorder and multimorbidity, and whether addressing the identified potential mediators in people with eating disorders can reduce multimorbidity.

## Introduction

When two or more physical conditions exist simultaneously in an individual, this condition is called physical multimorbidity [1]. Multimorbidity is an important risk concept as it is associated with adverse outcomes such as lower quality of life [2], unmet need, sickness days, reduced perceived health status [3], and higher risk for premature mortality [4]. In addition, a recent systematic review found that multimorbidity is associated with increased total healthcare costs including hospital costs, care transition costs, and costs for primary care, dental care, emergency department use, and hospitalizations [5]. Multimorbidity has a global prevalence of 42.4% [6], and in the UK, the prevalence of multimorbidity has been reported to be high (approximately 54% for those aged ≥ 65 years) and this is predicted to rise [7] mainly due to rapid ageing occurring in this country. Considering the profound impact of multimorbidity and its high prevalence, it is of utmost importance to identify groups at particularly high risk for this condition to inform targeted intervention and policy.

One group at potentially high risk for multimorbidity are those with eating disorders or wider abnormal eating behavior, as previous studies have shown that these conditions may increase risk for a variety of physical conditions such as osteoporosis [8], endocrine disorders, and kidney failure [9]. Eating disorders can be defined as a pathological relationship with food that leads to significant disruptions in a person's day to day life, and can lead to severe consequences, including premature mortality. These disorders have defined clinical criteria based on the Diagnostic and Statistical manual of mental disorders (DSM-5-TR) [10], or the International Classification of Diseases 11 (ICD-11) [11], and an estimated 70 million people worldwide suffer from eating disorders [12]. In particular, in the UK, it is thought that between 1.25 and 3.4 million people have an eating disorder [13]. A recent systematic review reported that the prevalence of eating disorders in adolescents and children was 22.4% [14], with some primary studies indicating higher prevalence rates for adults (~ 31%) [15], suggesting that this is a public health problem that needs to be addressed.

Eating disorders may lead to a higher risk for multimorbidity, for example, via low-grade inflammation [16]. For example, in the case of binge eating disorders, inflammation may be increased via overweight/obesity that often results in an increased production of free fatty acids, interleukin-6, tumor necrosis factor-α, hs-CRP, and rapid eating practices that result in elevated serum lipids [17, 18]. Indeed, inflammation can increase risk for multiple chronic conditions and multimorbidity, via alternations in immune function, for instance [19–23]. Moreover, eating disorders have been found to be associated with low bone mineral density [24], which in turn can increase risk for multiple chronic conditions (e.g., arthritis, osteopenia, osteoporosis) [25–27]. Finally, it is also possible that the association between eating disorders and multimorbidity is mediated by psychological factors (e.g., insomnia, stress, depression), lifestyle factors (e.g., smoking, alcohol consumption), and physical conditions (e.g., obesity, underweight) [28–41].

Despite the potential importance of eating disorders as a risk factor for multimorbidity, to the best of the authors’ knowledge, no research currently exists on the association between eating disorders and physical multimorbidity. Given this background, the aim of the present study was to investigate the association between eating disorder symptoms and possible eating disorder (≥ 2 eating disorder symptoms) in a sample of 7403 individuals aged ≥ 16 years who participated in the UK Adult Psychiatric Morbidity Survey. A further aim was to identify to what extent a variety of psychological, lifestyle, and physical factors may explain this association.

## Methods

### The survey

Cross-sectional data of the 2007 Adult Psychiatric Morbidity Survey were analyzed. Survey details are available elsewhere [42, 43]. In brief, this was a representative survey of the English adult population (aged ≥ 16 years) residing in private households. Fieldwork was undertaken by the National Center for Social Research, in conjunction with the University of Leicester in October 2006 to December 2007. A multistage stratified probability sampling design was used. The small user postcode address file was used as the sampling frame with postcode sectors as the primary sampling units. Addresses were stratified by region and by socio-economic groupings. A Kish grid was used to randomly select one respondent when there was more than one person in a household. Computer-assisted personal interviewing (CAPI) was used to obtain information from respondents. The survey response rate was 57%. To account for non-response and the probability of selection, sampling weights were generated so that the sample was representative of the English population. The Royal Free Hospital and Medical School Research Ethics Committee provided ethical approval for the survey with informed consent being obtained from all participants.

### Physical conditions and physical multimorbidity

Respondents were asked about 20 physical health conditions (cancer, diabetes, epilepsy, migraine, cataracts/eyesight problems, ear/hearing problems, stroke, heart attack/angina, high blood pressure, bronchitis/emphysema, asthma, allergies, stomach ulcer or other digestive problems, liver problems, bowel/colon problems, bladder problems/incontinence, arthritis, bone/back/joint/muscle problems, infectious disease, and skin problems). To be counted, conditions had to have been diagnosed by a doctor or other health professional and have been present in the previous 12 months. The number of physical conditions was summed and categorized as 0, 1, 2, 3, 4, and ≥ 5. Multimorbidity was defined as two or more physical conditions [44].

### Eating disorder symptoms and possible eating disorder

Five items from the SCOFF screening tool [45] were used to assess eating disorder symptoms and possible eating disorder, and consisted of whether, in the past year, the participant: had lost more than one stone (6.35 kg) in 3 months (weight loss); had made him/herself vomit because he/she felt uncomfortably full (self-sick for feeling full); worried that he/she had lost control over how much he/she eats (uncontrolled eating); believed to be fat when others said that he/she was too thin (self-perceived fatness); and thought that food dominated his/her life (food dominance). An affirmative reply to at least two items was considered a positive screen, and therefore classified as ‘possible eating disorder’ [45]. Although the SCOFF questionnaire is not a diagnostic instrument, it has been found to have high specificity (87–94%) and sensitivity (84–100%) in accordance with clinical diagnoses [45–48].

### Mediators

The potential mediators or influential factors in the association between possible eating disorder and physical multimorbidity considered in this study were insomnia, depression, anxiety disorder, alcohol dependence, lifetime smoking, perceived stress, obesity, and underweight.

Insomnia was defined as fulfilling all the following three criteria: (i) problems trying to get to sleep or getting back to sleep (if had woken up) in the past month; (ii) had problems with sleep on at least 4 nights in the past 7 nights in addition to (iii) taking at least 1 h trying to get to sleep on the night with least sleep [49]. The Clinical Interview Schedule Revised (CIS-R), which can be administered by lay interviewers, was used to generate ICD-10 diagnoses of depressive episode and anxiety disorder (generalized anxiety disorder, panic disorder, phobia, obsessive–compulsive disorder) in the prior week [50]. Alcohol consumption was assessed with the Alcohol Use Disorders Identification Test (AUDIT) [51]. Respondents whose AUDIT score was 10 or above were also assessed for alcohol dependence. This was done with the Severity of Alcohol Dependence Questionnaire (SADQ-C) [52], with a score of 4 or more (out of 60) being used to establish past 6-month alcohol dependence. Lifetime smoking referred to answering affirmatively to the question “Have you ever smoked a cigarette?” Participants were asked if their tasks at work and home very stressful with answer options “Most of the time”, “Usually”, “Occasionally”, and “Not at all”. Body mass index (BMI) was calculated as weight in kilograms divided by height in meters squared based on self-reported weight and height. Using the standard WHO definition, obesity referred to BMI ≥ 30 kg/m^2^, and underweight BMI < 18.5 kg/m^2^.

### Control variables

The control variables included sociodemographic variables (i.e., age, sex, education, ethnicity). Education referred to presence or absence of qualification (degree, non-degree, A-level, GCSE, other), and ethnicity was dichotomized into White British or other.

### Statistical analysis

The analyses were done with Stata version 14.2 (Stata Corp LP, College Station, Texas). The difference in sample characteristics between those with and without possible eating disorder was tested by Chi-squared tests except for age which was a continuous variable (Student’s *t*-test). Multivariable logistic regression analysis was done to assess the association between individual eating disorder symptoms or possible eating disorder (exposures) and individual physical conditions or physical multimorbidity (outcome), while adjusting for age, sex, education and ethnicity. Analyses with individual eating disorder symptoms as the exposure were also adjusted for the presence of ≥ 2 individual eating disorder symptoms as different eating disorder symptoms tend to co-exist. We also tested for interaction by sex in the association between the individual eating disorder symptoms or possible eating disorder and physical multimorbidity by including the product terms of these conditions and sex in the model but since preliminary analysis showed that there is no significant interaction, the analyses were not stratified by sex. Finally, in order to gain an understanding on the extent to which various factors (i.e., insomnia, depression, anxiety disorder, alcohol dependence, lifetime smoking, perceived stress, obesity, underweight) may explain the relation between possible eating disorder and physical multimorbidity, mediation analysis using the stata *khb* (Karlson Holm Breen) command in Stata was conducted [53]. This method can be applied in logistic regression models and decomposes the total effect of a variable into direct and indirect effects. Using this method, the percentage of the main association explained by the mediator can also be calculated (mediated percentage). Each potential mediator was included in the model individually, with the exception of the model which included all the potential mediators simultaneously. The mediation analysis controlled for sex, age, education, and ethnicity. In order to generate nationally representative estimates, in all analyses, the sample weighting and the complex study design were taken into account. Odds ratios (OR) and their 95% confidence intervals (95%CI) are reported. The level of statistical significance was set at *p* < 0.05.

## Results

A total of 7403 individuals aged ≥ 16 years were included in the analysis. The prevalence of possible eating disorder was 6.4%, while the prevalence of the individual eating disorder symptoms were: weight loss 10.8%; self-sick for feeling full 3.0%; uncontrolled eating 7.3%; self-perceived fatness 5.6%; food dominance 3.6%. The prevalence of physical multimorbidity was 35.1%. The sample characteristics are provided in Table 1. The mean (SD) age of the sample was 46.3 (18.6) years and 48.6% were males. People with possible eating disorder were significantly more likely to be younger, females, and have higher levels of education. Furthermore, they were also significantly more likely to have sleep problems, depression, anxiety, alcohol dependence, higher levels of perceived stress, and obesity.

**Table 1:** Sample characteristics (overall and by possible eating disorder)

Characteristic		Overall	Possible eating disorder		*p*-value
			No	Yes	
Age (years)	Mean (SD)	46.3 (18.6)	47.0 (18.6)	35.8 (14.2)	< 0.001
Sex	Male	48.6	50.1	26.5	< 0.001
	Female	51.4	49.9	73.5	
Qualification	No	23.9	24.5	14.3	< 0.001
	Yes	76.1	75.5	85.7	
Ethnicity	British White	85.2	85.4	82.6	0.206
	Other	14.8	14.6	17.4	
Insomnia	No	86.3	87.4	70.0	< 0.001
	Yes	13.7	12.6	30.0	
Depression	No	97.0	97.7	88.1	< 0.001
	Yes	3.0	2.3	11.9	
Anxiety	No	93.3	94.6	74.7	< 0.001
	Yes	6.7	5.4	25.3	
Alcohol dependence	No	94.1	94.7	85.1	< 0.001
	Yes	5.9	5.3	14.9	
Lifetime smoking	No	34.7	34.8	33.8	0.707
	Yes	65.3	65.2	66.2	
Perceived stress	Most of the time	5.7	5.1	14.2	< 0.001
	Usually	7.0	6.5	14.1	
	Occasionally	47.9	47.5	54.0	
	Not at all	39.4	40.8	17.7	
Obesity	No	82.3	83.1	70.2	< 0.001
	Yes	17.7	16.9	29.8	
Underweight	No	97.5	97.6	97.1	0.585
	Yes	2.5	2.4	2.9	

*SD* standard deviation

Data are % unless otherwise stated

Possible eating disorder referred to SCOFF screen-positive

*p*-value was calculated by Chi-squared tests except for age (Student’s *t*-test)

**Fig. 1:**
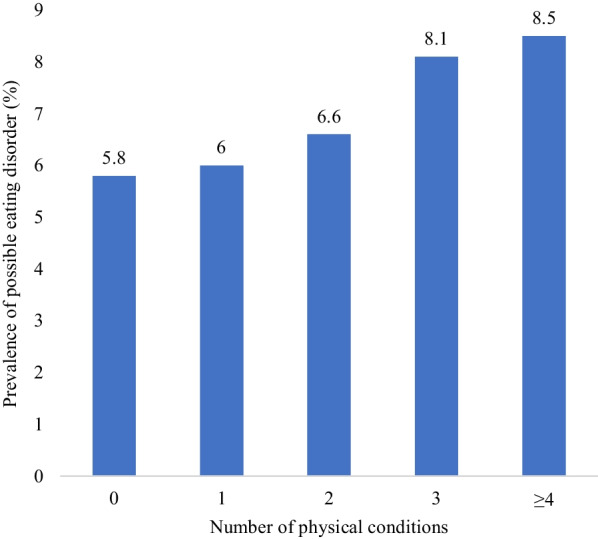
Prevalence of possible eating disorder by number of physical conditions

The prevalence of possible eating disorder increased linearly with increasing number of physical conditions (Fig. 1). Specifically, the prevalence was 5.8% among those with no physical conditions but this increased to 8.5% among those with ≥ 4 physical conditions. In terms of the individual physical conditions, possible eating disorder was significantly associated with higher odds for allergy, arthritis, asthma, bladder problems/incontinence, problems with back/bone/joint/muscle, bowel/colon problems, cancer, cataracts/eyesight problems, infectious diseases, liver problems, migraines/frequent headaches, skin problems, and stomach ulcer or other digestive problems (Table 2). Next, in terms of the individual eating disorder symptoms, weight loss was significantly associated with bowel/colon problems, cancer, cataracts/eyesight problems, heart attack/angina, high blood pressure, liver problems, migraine or frequent headaches, and stomach ulcer or other digestive problems (Appendix Table 5). Furthermore, uncontrolled eating was significantly associated with higher odds for asthma, bladder problems/incontinence, bowel/colon problems, diabetes, and high blood pressure, and self-perceived fatness with asthma, bowel/colon problems, skin problems, and stroke, while food dominance was significantly associated only with arthritis and liver problems.

**Table 2:** Association between possible eating disorder and individual physical condition (outcome) estimated by multivariable logistic regression

Physical condition	OR	[95%CI]
Allergy	1.47*	[1.08,2.02]
Arthritis	1.60**	[1.19,2.14]
Asthma	1.63**	[1.18,2.23]
Bladder problems/incontinence	2.18**	[1.26,3.77]
Bone, back, joint or muscle problems	1.38**	[1.09,1.74]
Bowel/colon problems	2.19***	[1.44,3.34]
Bronchitis/emphysema	1.81	[0.98,3.34]
Cancer	3.70**	[1.56,8.76]
Cataracts/eyesight problems	1.52**	[1.13,2.06]
Diabetes	1.61	[0.93,2.78]
Ear/hearing problems	1.40	[0.87,2.26]
Epilepsy/fits	2.36	[0.97,5.69]
Heart attack/angina	1.60	[0.73,3.52]
High blood pressure	1.29	[0.92,1.80]
Infectious disease	6.77***	[3.04,15.06]
Liver problems	2.40*	[1.07,5.39]
Migraine or frequent headaches	1.84***	[1.33,2.54]
Skin problems	1.52**	[1.13,2.04]
Stomach ulcer or other digestive problems	2.14***	[1.46,3.14]
Stroke	2.81	[0.52,15.08]

*OR* odds ratio, *CI* confidence interval

Models are adjusted for age, sex, education, and ethnicity

Possible eating disorder referred to SCOFF screen-positive

*p < 0.05, **p < 0.01, ***p < 0.001

Weight loss (OR = 1.64; 95%CI = 1.37–1.97), uncontrolled eating (OR = 1.87; 95%CI = 1.34–2.60), self-perceived fatness (OR = 1.69; 95%CI = 1.28–2.23), and possible eating disorder (OR = 2.11; 95%CI = 1.67–2.67) were associated with significantly higher odds for physical multimorbidity, but self-sick for feeling full and food dominance were not (Table 3). Mediation analysis showed that anxiety disorder explained the largest proportion of the association between possible eating disorder and physical multimorbidity (mediated percentage 26.3%), followed by insomnia (21.8%), perceived stress (13.4%), depression (13.1%), obesity (13.0%), and alcohol dependence (4.3%) (Table 4). These factors collectively explained 57.1% of the association.

**Table 3:** Association between individual eating disorder symptoms or possible eating disorder and physical multimorbidity (outcome) estimated by multivariable logistic regression

	OR	95%CI
Weight loss	1.64***	[1.37,1.97]
Self-sick for feeling full	1.32	[0.89,1.94]
Uncontrolled eating	1.87***	[1.34,2.60]
Self-perceived fatness	1.69***	[1.28,2.23]
Food dominance	1.13	[0.77,1.67]
Possible eating disorder	2.11***	[1.67,2.67]

*OR* odds ratio, *CI* confidence interval

Models are adjusted for age, sex, education, and ethnicity. Analyses with the individual eating disorders as the exposure are also adjusted for presence of ≥ 2 eating disorder symptoms

The definitions of eating disorder symptoms and possible eating disorder were: weight loss (had lost more than 6.35 kg in 3 months); self-sick for feeling full (had made him/herself be sick because he/she felt uncomfortably full); uncontrolled eating (worried he/she had lost control over how much he/she eats); self-perceived fatness (believed to be fat when others said that he/she was too thin); food dominance (thought that food dominated his/her life); possible eating disorder (SCOFF screen-positive)

Physical multimorbidity referred to ≥ 2 physical conditions

***p < 0.001

**Table 4:** Mediators in the association between possible eating disorder and physical multimorbidity (outcome)

Mediator	Effect	OR [95%CI]	*p*-value	Mediated %
Insomnia	Total	2.12 [1.66,2.70]	< 0.001	21.8
	Direct	1.80 [1.41,2.30]	< 0.001	
	Indirect	1.18 [1.12,1.24]	< 0.001	
Depression	Total	2.11 [1.66,2.68]	< 0.001	13.1
	Direct	1.92 [1.51,2.44]	< 0.001	
	Indirect	1.10 [1.06,1.15]	< 0.001	
Anxiety disorder	Total	2.11 [1.66,2.69]	< 0.001	26.3
	Direct	1.74 [1.36,2.22]	< 0.001	
	Indirect	1.22 [1.15,1.29]	< 0.001	
Alcohol dependence	Total	2.11 [1.67,2.66]	< 0.001	4.3
	Direct	2.04 [1.62,2.58]	< 0.001	
	Indirect	1.03 [1.00,1.06]	0.023	
Lifetime smoking	Total	2.11 [1.67,2.67]	< 0.001	NA
	Direct	2.09 [1.66,2.64]	< 0.001	
	Indirect	1.01 [1.00,1.02]	0.122	
Perceived stress	Total	2.11 [1.66,2.68]	< 0.001	13.4
	Direct	1.91 [1.50,2.43]	< 0.001	
	Indirect	1.10 [1.06,1.15]	< 0.001	
Obesity	Total	2.15 [1.68,2.76]	< 0.001	13.0
	Direct	1.95 [1.52,2.50]	< 0.001	
	Indirect	1.11 [1.06,1.15]	< 0.001	
Underweight	Total	2.14 [1.68,2.72]	< 0.001	NA
	Direct	2.14 [1.68,2.72]	< 0.001	
	Indirect	1.00 [0.99,1.01]	0.621	
All mediators	Total	2.19 [1.68,2.84]	< 0.001	57.1
	Direct	1.40 [1.07,1.83]	0.014	
	Indirect	1.56 [1.42,1.72]	< 0.001	

*OR* odds ratio, *CI* confidence interval

Models are adjusted for age, sex, education, and ethnicity

Possible eating disorder referred to SCOFF screen-positive

Mediated percentage was only calculated in the presence of a significant indirect effect

The mediators were included individually in the model, except for the model which included all mediators simultaneously

## Discussion

### Main findings

In the present study including a large representative sample of adults aged ≥ 16 years from the UK, possible eating disorder was significantly associated with a more than twofold increased odds for physical multimorbidity, while individual eating disorder symptoms of weight loss, uncontrolled eating, and self-perceived fatness were significantly associated with 1.64–1.87 times higher odds for physical multimorbidity. The factors which were most important in the association between possible eating disorder and physical multimorbidity were anxiety disorder and insomnia, which explained more than 20% of the association. Factors such as perceived stress, depression, obesity, and alcohol dependence also explained this association but to a lesser extent. To the best of the authors’ knowledge, this is the first study to investigate the association between eating disorder symptoms or possible eating disorder and physical multimorbidity, and also the first to quantify the extent to which this association may be explained by a variety of factors.

### Interpretation of the findings

There are several plausible pathways that likely explain the relationship between possible eating disorder and physical multimorbidity. In our study, anxiety disorder, insomnia, perceived stress, depression, obesity, and alcohol dependence were identified as potential influential factors in this association, with anxiety disorder and insomnia being particularly important. Literature suggests that eating disorders and anxiety disorders frequently co-occur, and it is likely that the relationship is bi-directional [34]. Common neurobiological, genetic, and psychological elements may be implicated in this co-existence [34]. Furthermore, eating disorders may lead to anxiety, perceived stress, and depression by, for example, body dissatisfaction, low self-esteem and uncontrollability of the situation [54]. These psychological factors, in turn, may increase risk for physical multimorbidity via elevated inflammation and immune dysregulation [55]. In terms of insomnia, eating disorders may increase risk for insomnia due to malnutrition and orexin. Indeed, orexin, a neuropeptide released from the hypothalamus, is involved in the regulation of both sleep–wake and appetite; increased orexin signaling promotes greater wakefulness and feeding. It has been posited that levels of orexin are increased during the hunger state to promote wakefulness and incite the body to search for food [56]. In turn, insomnia may increase risk for multimorbidity through dysfunction of the inflammatory system [57].

Obesity was another important influential factor identified in the present study. Some eating disorders, such as binge eating disorder, likely cause obesity owing to large consumptions of high energy dense foods, and obesity is likely to be a key risk factor for multimorbidity by increasing risk for hypertension, unfavorable cholesterol profile, dysfunction in the regulation of blood sugar levels, and dysfunction of the inflammatory system [58]. Finally, alcohol dependence also explained a small proportion of the association between eating disorders and physical multimorbidity. Those with eating disorders may turn to alcohol as a coping mechanism, thereby making them susceptible to alcohol dependence [35]. In turn, alcohol dependence is known to increase risk for a variety of physical diseases including cardiovascular disease, liver disease, and malignant neoplasms [59].

It is also important to note that all the influential factors included in our study collectively explained less than 60% of the association between possible eating disorder and physical multimorbidity. It is possible that there are unmeasured mechanisms such as inflammation [16], change in bone structure [24], and malnutrition.

Interestingly, weight loss, uncontrolled eating, and self-perceived fatness were associated with higher odds for physical multimorbidity but self-sick for feeling full and food dominance were not. It is likely that extreme weight loss and uncontrolled eating are associated with physical multimorbidity as both likely promote inflammatory dysregulation, and perceived fatness likely promotes physical multimorbidity via stress, anxiety, and depression. In the case of self-sick and food dominance, it is possible that these two constructs alone have little impact on physiological and psychological mechanisms that drive chronic disease and physical multimorbidity.

### Strengths and limitations

The analysis of a large representative sample of English adults and validated measures are clear strengths of the present study. However, findings must be interpreted in light of the study’s limitations. First, the study was cross-sectional in nature, and thus, the direction of the association cannot be established. For example, weight loss can also be the consequence of the physical condition (e.g., cancer). Second, the variables included in the study were based on self-report, potentially introducing recall and social desirability bias into the findings. Third, there was no data on physical activity in the dataset despite this factor being associated with both eating disorder and multimorbidity. Thus, we were unable to examine the influence of this factor in the association between eating disorder and multimorbidity. Furthermore, although the SCOFF questionnaire does have favorable validity, it is not a diagnostic tool. Thus, future research should aim to use clinician diagnosed eating disorders to confirm these findings. Also, we used BMI to determine weight category. However, BMI may not be the optimal measure to employ to measure body mass since it does not distinguish between fat mass and fat-free mass, and thus, may categorize an individual with a high level of fat-free mass as overweight or obese. Different measures of adiposity (e.g., waist circumference, bioimpedance) may yield differing mediating effects. Next, while we did have data on underweight which could be considered a proxy of malnutrition, we did not have data on malnutrition per se, despite the fact that eating disorders are associated with a high risk for malnutrition, and malnutrition can increase risk for multiple physical diseases [60, 61]. For example, pathophysiologic changes of the esophagus, stomach, and intestines develop with malnutrition, which can lead to gastrointestinal conditions such as celiac disease and inflammatory bowel disease [62]. Finally, mediation and confounding are identical from a statistical point of view, and can be distinguished only on conceptual grounds [63]. Thus, although the influential variables that were assessed in this study could be conceptualized as mediators, the mediated percentage estimated for each potential influential factor in our study could also be due to confounding.

### Clinical implications and areas for future research

The results of our study suggest that people with possible eating disorder may be at high risk for physical multimorbidity. Clinicians should be aware of this so that people with eating disorders can be screened for physical conditions, and have them treated, or vice versa. Furthermore, our study results suggest that addressing the identified potential mediators in the present study among those with eating disorders may help in the prevention of physical multimorbidity. Indeed, a well-established and lasting treatment for depression, anxiety, alcohol dependence, perceived stress, and sleep problems is cognitive-behavioral therapy, which focuses on identifying, understanding, and changing thinking and behavior patterns [64–67]. Importantly, cognitive-behavioral therapy can also be utilized as a treatment approach for eating disorders per se [68]. Interestingly, in the present study, even individual eating disorder symptoms (i.e., weight loss, uncontrolled eating, self-perceived fatness) were associated with physical multimorbidity. Thus, these findings suggest that the detection of individual symptoms alone could identify people at high risk for multimorbidity. Finally, studies of longitudinal design on eating disorders and physical multimorbidity are necessary to provide a better understanding of temporal associations, causality, and the underlying mechanisms.

## Conclusion

In this large sample of UK adults, it was observed that possible eating disorder was significantly associated with physical multimorbidity and that this association was partly explained by anxiety disorder, insomnia, perceived stress, depression, obesity, and alcohol dependence. While temporal associations and causality could not be assessed in our cross-sectional study, the mere co-existence of possible eating disorder and physical multimorbidity could complicate the management of these conditions, while they can also mutually influence each other and lead to worse clinical outcomes. Future longitudinal studies are warranted to understand potential causality and the underlying mechanisms, and also whether addressing the identified potential mediators in people with eating disorders can lead to a lower risk for physical multimorbidity Table 5.

**Table 5:** Association between individual eating disorder symptoms and individual physical conditions (outcome) estimated by multivariable logistic regression

Physical condition	Weight loss	Self-sick for feeling full	Uncontrolled eating	Self-perceived fatness	Food dominance
Allergy	1.05	0.57	1.45	0.80	0.99
	[0.80,1.37]	[0.31,1.04]	[0.98,2.15]	[0.55,1.17]	[0.57,1.72]
Arthritis	1.22	1.23	1.46	0.96	1.79*
	[0.96,1.57]	[0.57,2.66]	[0.95,2.26]	[0.62,1.49]	[1.04,3.08]
Asthma	1.34	0.90	1.85*	1.57*	1.18
	[1.00,1.79]	[0.54,1.49]	[1.14,3.01]	[1.07,2.31]	[0.70,1.97]
Bladder problems/incontinence	1.42	1.54	3.06**	0.78	1.30
	[0.91,2.19]	[0.62,3.84]	[1.34,6.98]	[0.38,1.62]	[0.47,3.56]
Bone, back, joint or muscle problems	1.14	0.92	1.19	1.13	0.85
	[0.93,1.40]	[0.60,1.40]	[0.91,1.57]	[0.84,1.51]	[0.56,1.28]
Bowel/colon problems	2.31***	1.18	1.79*	1.65*	1.11
	[1.75,3.05]	[0.52,2.69]	[1.03,3.09]	[1.03,2.64]	[0.59,2.09]
Bronchitis/emphysema	1.92	1.41	0.98	1.69	1.95
	[0.87,4.27]	[0.43,4.61]	[0.25,3.84]	[0.38,7.49]	[0.73,5.22]
Cancer	1.32*	1.22	1.05	1.23	0.83
	[1.05,1.66]	[0.74,2.01]	[0.69,1.61]	[0.88,1.72]	[0.44,1.54]
Cataracts/eyesight problems	1.58*	1.02	0.80	1.11	1.92
	[1.10,2.27]	[0.38,2.75]	[0.41,1.56]	[0.55,2.23]	[0.95,3.90]
Diabetes	1.10	1.38	1.87*	1.05	1.87
	[0.78,1.54]	[0.66,2.87]	[1.02,3.42]	[0.53,2.07]	[0.98,3.57]
Ear/hearing problems	1.60	1.49	1.50	1.54	1.38
	[0.94,2.70]	[0.50,4.44]	[0.71,3.15]	[0.68,3.49]	[0.45,4.24]
Epilepsy/fits	1.19	1.89	1.17	1.24	0.95
	[0.50,2.81]	[0.69,5.16]	[0.36,3.74]	[0.42,3.60]	[0.24,3.83]
Heart attack/angina	2.37***	0.50	0.70	0.76	1.03
	[1.44,3.88]	[0.11,2.19]	[0.33,1.48]	[0.28,2.03]	[0.34,3.12]
High blood pressure	1.34*	1.47	1.50*	1.10	1.42
	[1.05,1.73]	[0.82,2.64]	[1.06,2.11]	[0.78,1.55]	[0.84,2.39]
Infectious disease	2.31	1.34	0.48	1.76	0.38
	[0.97,5.50]	[0.36,4.92]	[0.09,2.49]	[0.58,5.30]	[0.09,1.52]
Liver problems	2.95**	1.55	0.98	0.61	4.88*
	[1.38,6.28]	[0.33,7.16]	[0.49,1.94]	[0.17,2.20]	[1.40,17.06]
Migraine or frequent headaches	1.36*	1.04	1.32	1.51*	0.95
	[1.03,1.79]	[0.57,1.90]	[0.85,2.05]	[1.00,2.28]	[0.59,1.51]
Skin problems	1.01	0.69	1.39	1.13	0.85
	[0.78,1.31]	[0.40,1.22]	[0.87,2.21]	[0.76,1.68]	[0.53,1.39]
Stomach ulcer or other digestive problems	1.75**	1.48	1.53	1.31	1.61
	[1.26,2.45]	[0.74,2.98]	[0.81,2.86]	[0.80,2.15]	[0.86,3.01]
Stroke	1.65	1.00	0.12	3.88*	0.80
	[0.52,5.22]	[1.00,1.00]	[0.01,1.53]	[1.08,13.86]	[0.02,30.48]

Data are odds ratio [95% confidence interval]

Models are adjusted for age, sex, education, ethnicity, and presence of ≥ 2 eating disorder symptoms

The definitions of eating disorder symptoms were: weight loss (had lost more than 6.35 kg in 3 months); self-sick for feeling full (had made him/herself be sick because he/she felt uncomfortably full); uncontrolled eating (worried he/she had lost control over how much he/she eats); self-perceived fatness (believed to be fat when others said that he/she was too thin); food dominance (thought that food dominated his/her life);

*p < 0.05, ** *p* < 0.01, *** *p* < 0.001

## Data Availability

The data that support the findings of this study are available from the corresponding author upon reasonable request.

## Appendix

See Table 5
